# Abdominal Pain, an Atypical Presenting Symptom of Granulomatosis with Polyangiitis

**DOI:** 10.7759/cureus.7864

**Published:** 2020-04-27

**Authors:** Krishna Desai, Merin Jose, Osakpolor Ogbebor

**Affiliations:** 1 Internal Medicine, Terna Medical College, Mumbai, IND; 2 Internal Medicine, Saint Peters University Hospital, New Brunswick, USA; 3 Internal Medicine, Allegheny General Hospital, Pittsburgh, USA

**Keywords:** anca associated vasculitis, c anca, rpgn

## Abstract

Granulomatosis with polyangiitis (GPA) formerly known as Wegener’s granulomatosis, is an anti-neutrophil cytoplasmic autoantibody (ANCA)-associated vasculitis (AAV). It is an uncommon disease with an estimated prevalence of 3 in 100,000 individuals with an equal distribution in both sexes. It is characterized by necrotizing granulomatous vasculitis that primarily affects the upper and lower respiratory tracts and the kidneys.

Our patient's initial presentation was abdominal pain with no typical pulmonary or renal manifestations. Along the course of her hospitalization, she had multiple episodes of drop in hemoglobin and a steady increase in serum creatinine which was thought to be due to IV contrast nephropathy. With this case, we project the need for a high index of clinical suspicion to make an early diagnosis, especially in patients with atypical symptoms such as abdominal pain, and acknowledge the fact that IV contrast can possibly act as a second hit in underlying GPA, unmasking the active renal symptoms of the disease.

## Introduction

Granulomatosis with polyangiitis (GPA) is a multisystem inflammatory, small vessel disease that can affect any organ system, but most frequently targets the upper and lower respiratory tracts and the kidneys. Most commonly, the prodromal symptoms precede organ involvement, and may persist for weeks to months before presenting with an organ-specific manifestation. Antineutrophil cytoplasmic autoantibody (ANCA)-associated vasculitis (AAV) include GPA, microscopic polyangiitis (MPA), renal-limited vasculitis and eosinophilic granulomatosis with polyangiitis (EGPA, Churg-Strauss). They all have a very close resemblance in presentation and appear similar on kidney biopsy, i.e. crescentic, focal necrotizing, pauci-immune glomerulonephritis, attributing to the challenges in reaching an accurate diagnosis on initial presentation [1]. Abdominal symptoms and gastrointestinal (GI) complications though seldom, can be the main presenting symptom of the disease; as with our patient, who presented with abdominal pain weeks before having any typical identifiable symptoms of GPA. The objective of our case report is to emphasize on having a focused approach in identifying atypical symptoms at initial presentation, essential to expedite an early diagnosis to abate the progression of an otherwise fatal disease. 

## Case presentation

A 63-year-old Hispanic female presented to our hospital with chronic abdominal pain of six weeks. She initially presented to a nearby hospital with right upper quadrant abdominal pain and an assessment of cholecystitis was made, as HIDA SCAN showed cystic duct obstruction. A cholecystectomy was planned, but her hospital stay was complicated by respiratory failure with presumed hospital-acquired pneumonia, escalating her care to the ICU and intubation. On recovery, she was discharged home, after 29 days of admission. Some 13 days after the discharge, she presented to our ED with abdominal pain, dyspnea, and fever.

**Figure 1:**
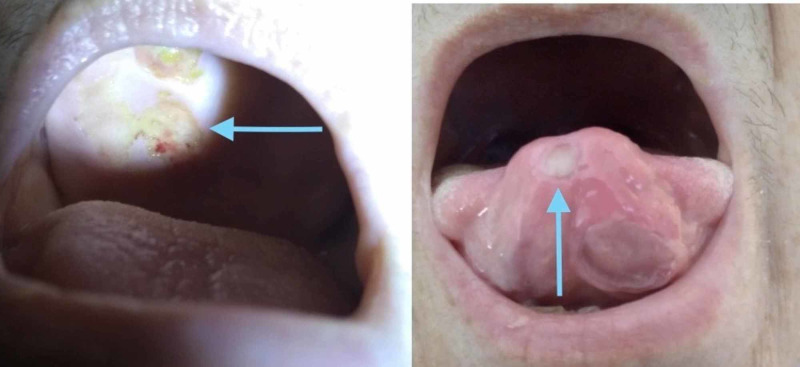
Mouth ulcers on the tongue and the roof of the mouth.

Her past medical history was only significant for hypertension and multiple abdominal reconstruction surgeries (including a splenectomy) following a motor vehicle accident, five years back. Her vitals on presentation were: blood pressure was 103/54 mmHg. She had a brief period of hypotension to 81/41 mmHg, within 24 h of admission, which responded to adequate fluid resuscitation. Her temperature was 99.7°F on presentation, with episodes of fever observed with a maximum temperature of 102.6°F within 24 h. Her oxygen saturation was 93% on room air and she desaturated to 88% on ambulation, requiring her to be started on oxygen therapy - 2L through a nasal cannula. She had tenderness in the right upper quadrant and epigastric region of the abdomen on deep palpation. Breath sounds were clear bilaterally. No murmurs were appreciated. Ulcers were observed on the roof of the mouth and tongue (Figure 1).

Initial blood test showed a white cell count (WCC) of 12.5 cells/cubic millimeter with a neutrophilic predominance, hemoglobin of 9.0 g/dL, and platelet count of 389 x 103 cells/mL. The chemistry panel showed a sodium level of 132 mEq/L, blood urea nitrogen (BUN) of 19 mg/dL, creatinine of 1.04 mg/dL, and estimated glomerular filtration rate (eGFR) of 53 mL/min/1.73 m2. 

**Figure 2:**
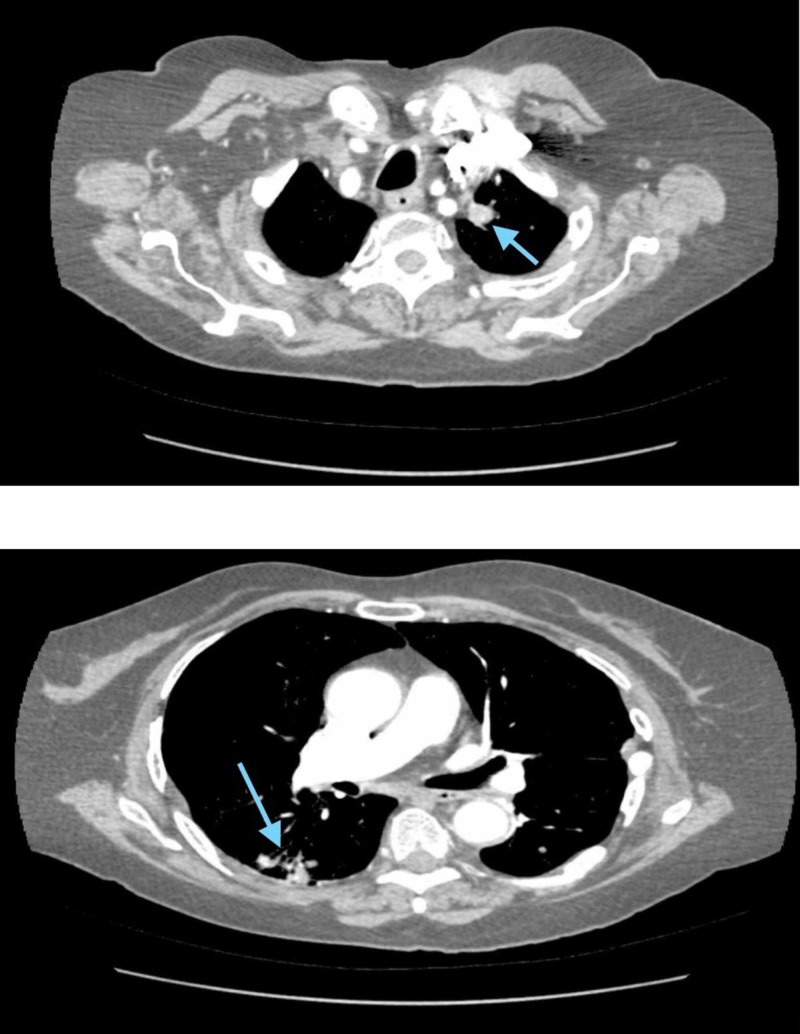
CT angiogram showing nodules in the left lung apex and posterior right lung.

A CT pulmonary angiogram (CTPA) was performed and was compared to CT scans she had during her most recent admission. The CTPA showed no evidence of pulmonary embolism. A stable left apical mass measuring 1.4 cm in diameter with interval development of multiple nodular densities within the right upper lobe, right lower lobe, and lingula segment of the left upper lobe and left lower lobe was observed (Figure 2).

**Figure 3:**
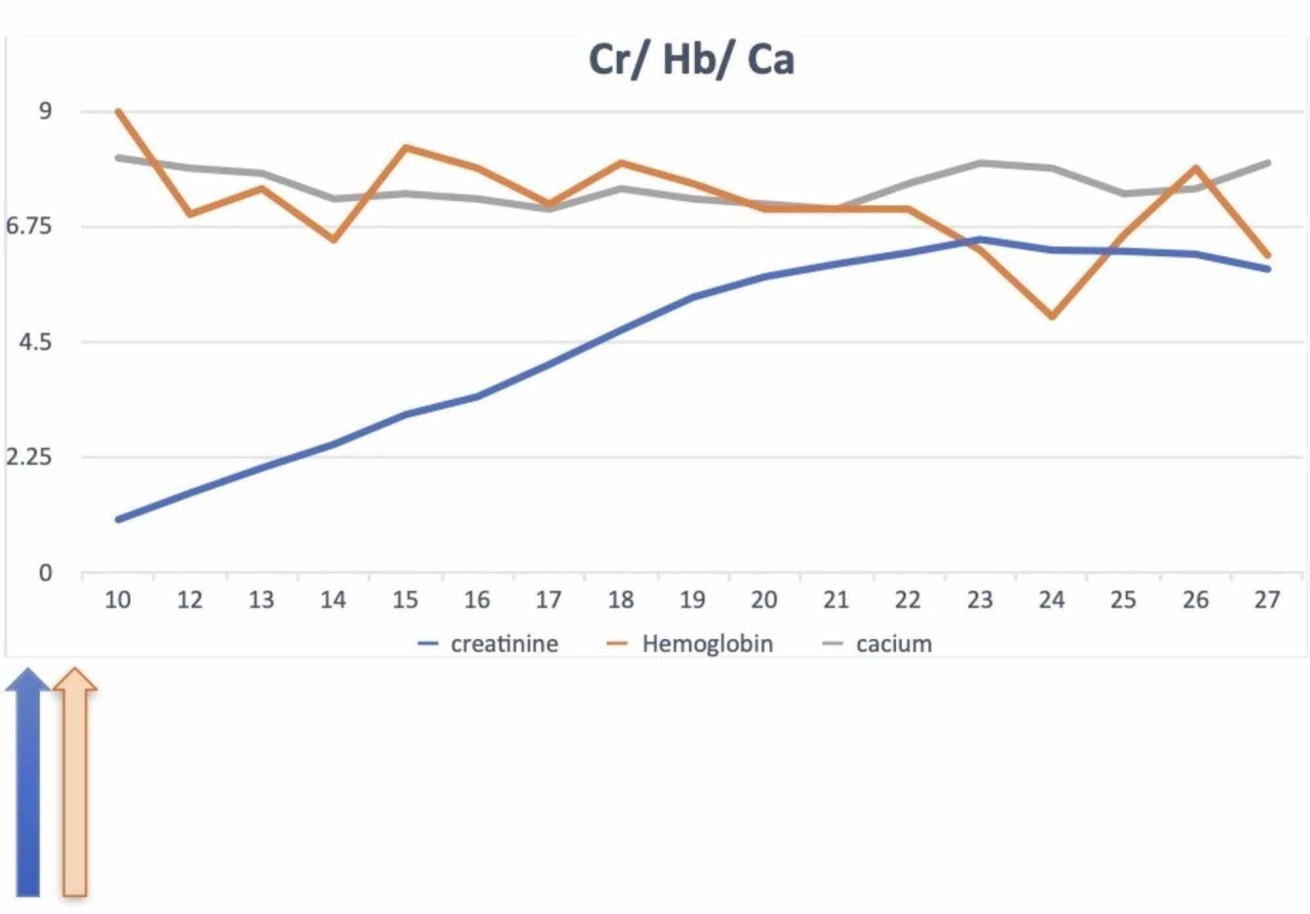
Trend of creatinine and hemoglobin levels.

**Figure 4:**
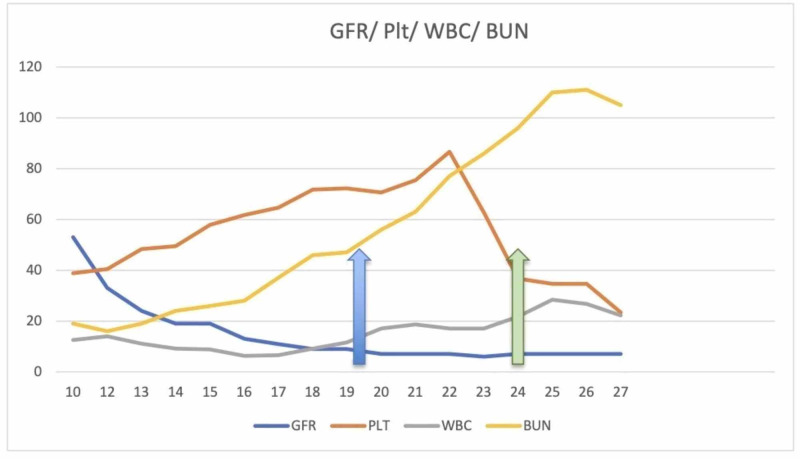
Trend of BUN, GFR, platelet, and WBC. BUN, blood urea nitrogen; GFR, glomerular filtration rate; WBC, white blood cell

The trends of the levels of the relevant labs during the hospital course is shown in Figures 3-4. 

As evident in the graph, she had multiple episodes of drop in hemoglobin, attributed to melena, which she developed during her admission. Of note, initial spike in creatinine was presumed to be due to IV contrast, and so, a major differential to consider with this presentation was, contrast nephropathy. It was observed that 48 h after the administration of the contrast for CTPA, there was a sequential rise in creatine. However, a fractional excretion of sodium (FeNa) of 1.4% was calculated from urine electrolytes. FeNa of 1.4% was consistent with an intrinsic kidney failure. This was further associated with proteinuria and hematuria. The CTPA showed multiple pulmonary nodules and cavitating lesion at the upper lobe, which caused the patient to be symptomatic. Barium study was normal. An echocardiogram was also normal, ruling out infective endocarditis and the possibility of septic emboli to the lungs. In view of her abdominal pain, the initial presenting symptom, a cholescintigraphy was performed, which ruled out cholecystitis.

The clinical features of pulmonary-renal syndrome and increasing creatinine, prompted further investigation. Anti-GBM, anti-nuclear antigen (ANA), creatinine kinase, C3, C4 complement, serum IgG, IgA and IgM, all were normal. Considering the clinical course of the patient and the sudden deterioration of her renal function post-contrast, we speculate IV contrast to act as a second hit to the underlying GPA, unmasking its typical renal involvement.

**Figure 5:**
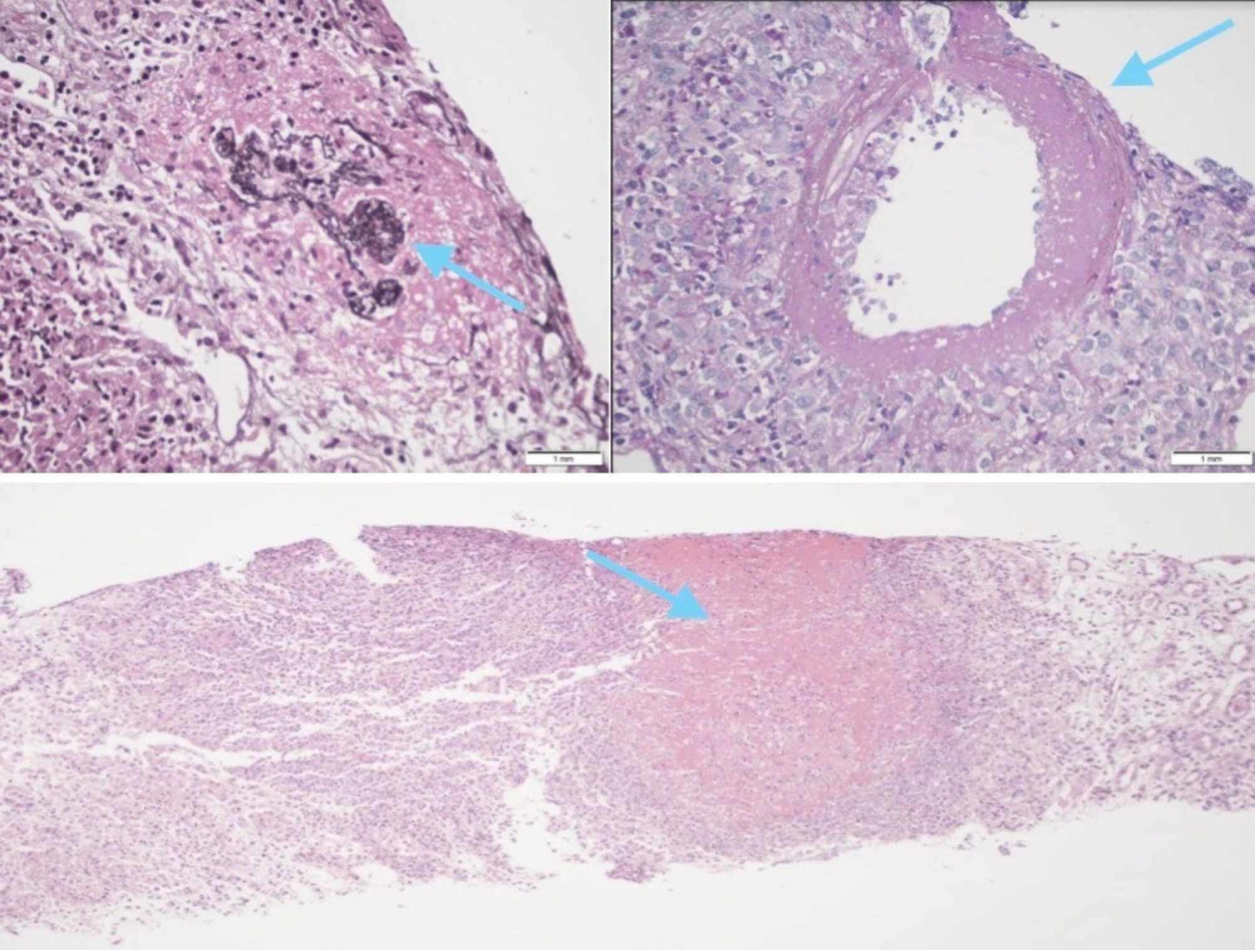
Histology showing acute necrotizing granulomatous interstitial inflammation, diffuse and severe geographic necrosis, and multifocal necrotizing glomerulonephritis and arteritis.

Serology was positive for C-ANCA with a titre of 1:640. A kidney biopsy showed acute necrotizing granulomatous interstitial inflammation, diffuse and severe with geographic necrosis and multifocal necrotizing glomerulonephritis and arteritis (Figure 5).

The patient had a convoluted hospital course and her management changed as the diagnosis revealed itself. On admission, she was initially started on antibiotics for suspected sepsis. This was later stopped on the third day of hospitalization, as she was no longer febrile and was asymptomatic with no findings suggestive of an infective etiology for her symptoms. 

With rising creatinine, which did not respond to intravenous fluids, and with the suspicion of a possible vasculitis, she was started on methylprednisone 500 mg IV for three days and transitioned to oral prednisone 60 mg PO once daily which she received for eight days. During this period, the ANCA test was positive and the renal biopsy was done and the diagnosis of GPA was made on the 10th day of hospitalization. 

**Figure 6:**
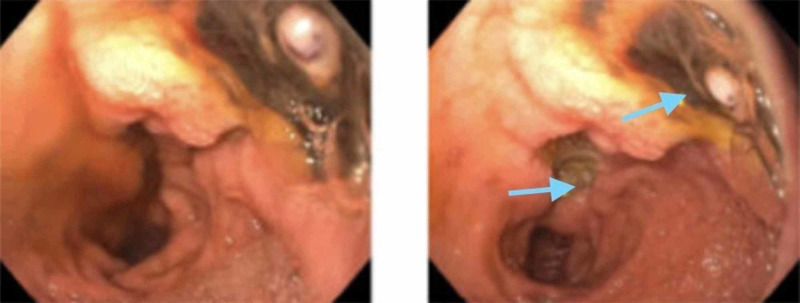
A 35-mm nonbleeding cratered duodenal ulcer with a visible vessel in the duodenal bulb on the posterior wall.

Although, planned to commence rituximab and a treatment course of plasmapheresis, this was put on hold, because she developed GI hemorrhage. Endoscopy showed a large 3.5 cm cratered nonbleeding duodenal ulcer containing visible vessel on the posterior wall of the duodenal bulb with multiple ulcers (Figure 6).

Further episodes of GI bleed occurred for which she required multiple blood transfusions and radiology-guided arterial embolization. 

Once stabilized, she was started on cyclophosphamide. She had seven sessions of plasmapheresis over a period of 14 days. She had one session of hemodialysis, in view of respiratory failure from fluid overload. She also had a thoracentesis for pleural effusion, in view of respiratory distress. A palliative team consult was also achieved, with the goals of care addressed. One month after admission, the patient decided not to pursue any further aggressive treatment including hemodialysis.

After starting on steroids the rate of increase in creatinine level plateaued and following the first dose of cyclophosphamide her creatinine level improved, as noted in the trend of the creatinine level in the graph. But overall her condition worsened due to multiple episodes of GI bleed, resulting in transfusion of almost 10 units of packed red blood cells. It was further complicated by acute hypoxic respiratory failure from fluid overload and pleural effusion. The patient died after five weeks of admission. 

## Discussion

Granulomatosis with polyangiitis is a progressive multisystem vasculitis of small arteries and veins. It is a rare disease and was found to have an estimated prevalence of 3 in 100,000 individuals with an equal distribution in both sexes [2-3]. The mean and median period from the onset of the symptoms to the diagnosis of GPA are 4.7 and 15 months, respectively. It is a known cause of pulmonary-renal syndrome, affecting the kidneys and respiratory tract. GI involvement is rare and this manifests as oral ulcers as well as GI bleeding [4]. In a study that evaluated 41 patients with GPA, only 2 (5%) had GI bleeding [5]. It is suggested that the presence of GI bleeding heralds a poor prognosis. The American College of Rheumatology Criteria for diagnosis requires the presence of two out of the following four criteria - 1) presence of nasal or oral inflammation (painful or painless oral ulcers, or purulent or bloody nasal discharge); 2) abnormal chest imaging showing nodules, fixed infiltrates, or cavities; 3) presence of abnormal urinary sediment (microscopic hematuria with or without red cell casts); and 4) granulomatous inflammation on biopsy of an artery or perivascular area. Although, the above criteria yields 88% sensitivity and 92% specificity, for making the diagnosis of GPA, it does not differentiate between GPA, MPA, or any nonvasculitic diseases which mimic GPA, making the diagnosis extremely challenging, even if it has a usual presentation. There is a revised diagnostic criteria being developed by the American College of Rheumatology/European League Against Rheumatism (ACR/EULAR) collaboration [6-7]. Despite this, there are several studies and case reports that have emphasized the challenges in making a diagnosis and a high index of suspicion is required [8]. As seen in this patient, who initially presented to the hospital for abdominal pain, the diagnosis was incidentally unmasked due to rapidly deteriorating renal function, initially attributed to contrast-based radiological studies. The timeline in our case fits with contrast nephropathy. However, this usually has a FeNa less than 1%, at least in the early stages. Also, the serum creatinine was rising with an increment of 0.5 mg/dL/day, despite the reno-protective measures, indicating a rapidly progressive glomerulonephritis. This warranted a more detailed diagnostic approach, such as renal tissue biopsy and presence of ANCA. 

If the glomerulopathy is left untreated, it can precipitate end-stage renal disease. Serum creatinine and the number of normal glomeruli on biopsy are the best indicators of the prognosis [9]. C-ANCA is highly specific for GPA, especially if an active glomerulonephritis is present. But its absence does not exclude the disease. Therefore, tissue biopsy is the best confirmatory test for GPA [10].

Corticosteroids with either cyclophosphamide or in addition to plasmapheresis, are the best initial treatment regimen. Whereas, rituximab was equally potent, or maybe even superior in inducing remission, maybe because it causes B-cell depletion. It is also used in younger age group for salvaging the fertility. Adjunctive plasmapheresis is proven to be of great value in patients with rapidly progressive glomerulonephritis and creatinine values of >5.7 mg/dL, as in our patient, who had peaked the values of 6.5 mg/dL, but arrived with normal renal function on initial presentation [9].

## Conclusions

Abdominal symptoms and GI complications could be a presentation of GPA. A high index of suspicion for GPA should be present in patients with nonspecific or any atypical presenting complaints, especially those later accompanied with deteriorating renal function. With rapidly increasing creatinine levels, GPA should always remain as a potential differential, especially if there is evidence of pulmonary-renal syndrome. IV contrast can act as a second hit in patients with underlying GPA, for active renal disease to manifest itself. Multi-organ complaints with multi-organ pathology should not be dealt as two distinct entities, but rather can more often be explained as a single diagnosis. 
